# On the expanding roles of tRNA fragments in modulating cell behavior

**DOI:** 10.1093/nar/gkaa657

**Published:** 2020-09-05

**Authors:** Rogan Magee, Isidore Rigoutsos

**Affiliations:** Computational Medicine Center, Thomas Jefferson University, 1020 Locust Street, Philadelphia, PA 19107, USA; Computational Medicine Center, Thomas Jefferson University, 1020 Locust Street, Philadelphia, PA 19107, USA

## Abstract

The fragments that derive from transfer RNAs (tRNAs) are an emerging category of regulatory RNAs. Known as tRFs, these fragments were reported for the first time only a decade ago, making them a relatively recent addition to the ever-expanding pantheon of non-coding RNAs. tRFs are short, 16–35 nucleotides (nts) in length, and produced through cleavage of mature and precursor tRNAs at various positions. Both cleavage positions and relative tRF abundance depend strongly on context, including the tissue type, tissue state, and disease, as well as the sex, population of origin, and race/ethnicity of an individual. These dependencies increase the urgency to understand the regulatory roles of tRFs. Such efforts are gaining momentum, and comprise experimental and computational approaches. System-level studies across many tissues and thousands of samples have produced strong evidence that tRFs have important and multi-faceted roles. Here, we review the relevant literature on tRF biology in higher organisms, single cell eukaryotes, and prokaryotes.

## INTRODUCTION

By all accounts, next generation sequencing (NGS) has led to important discoveries while also enabling many practical applications in biology and medicine. Over the last decade, the discovery of new molecules with the help of NGS far outpaced the elucidation of the function of individual molecules. Due to current technical limitations, the functions of many individual molecules in these newly discovered categories elude us. Nonetheless, the decreasing cost and ready availability of NGS systems is allowing practitioners to draw conclusions about the roles of some of these new molecular classes as a whole, through well-designed experiments and analyses. Decidedly, one of the important conclusions reached by carrying out NGS *en masse* across many *like* samples is that these novel molecules do not result from degradation and that their production is regimented.

In this review, we focus on the short RNAs that are produced from tRNAs and are known as tRNA-derived fragments (tRFs) (1,2). The enumeration and characterization of tRFs accelerated greatly in the last few years, due in large part to the emergence of NGS systems-level approaches to molecular genetics (3). tRFs range in length from 16 to 35 nucleotides (nts) and are produced through cleavage of mature and premature tRNAs at various positions. These cleavages can occur essentially anywhere along the length of the tRNA, and are context-dependent, as demonstrated by a recent large-scale study of the samples in The Cancer Genome Atlas (TCGA) (4). Notably, as we will discuss below, the majority of tRFs overlap one another and are not adjacent, which suggests complex biogenesis processes (5).

## THE EARLY DAYS OF TRFS

### The earliest reports of tRNA fragments

The first tRFs were actually characterized during the race to determine the structure of tRNA (6) and the reader is referred to excellent historical perspective by Paul Schimmel (7). One such tRF, 19 nucleotides (nt) in length, from the tRNA^fMet^ of *E. coli* was shown to interact with the 30S ribosomal subunits (8). This fragment was initially identified when the entire structure of this initiator tRNA was solved (9). Around the same time, a 16 nt fragment of the yeast tRNA^Phe^ was shown to competitively inhibit phenylalanyl-tRNA synthetase activity at the 3′ CCA tail (necessary for amino acid charging) (10).

Initial evidence that these fragments may be biologically relevant came five years later. Work by Petrova *et al.* demonstrated that eight different snake exonuclease preparations led to the production of tRFs in a tRNA-tertiary-structure-specific manner (11). The team noted that some of the tRFs they observed on northern blots were likely further processed by additional endonucleases in the venom preparations. The fact that these species underwent multiple rounds of cleavage suggested that processing of tRNAs into specific smaller RNA fragments was a regimented process.

## TO STRESS OR NOT TO STRESS ABOUT IT?

### tRNA fragments and cellular stress

Several independent groups linked stress responses to tRNA cleavage as early as 2005. Lee and Collins reported that amino acid starvation in *T. thermophila* led to the accumulation of fragments that resulted from cleavage of the tRNA at the anticodon loop (12). These tRNA ‘halves’ were approximately 35 nt in length and arose from only some of the present tRNAs. Additionally, the 3′ ‘halves' lacked the -CCA tail modification that is necessary for amino acid charging (12). Similar findings were reported in A. *fumigatus* (13).

Related work showed that in *S. coelicolor* the cleavage of tRNAs did not depend on amino acid starvation but on nutrient availability in the growth media (14). The cleavage occurred during the bacteria's protective transformation to spore and hyphal structures under nutrient deprivation. Moreover, it was more prevalent among tRNAs from highly-used codons (14). Similar accumulation of 3′-halves was observed during encystation of *G*.*lamblia*, as well as under serum starvation (15).

A comprehensive screen that relied on northern blots provided strong evidence of consistent splitting of tRNAs through the anticodon loop into 5′- and 3′-halves (16). Further examination showed that oxidative stress, heat stress, UV stress, glucose starvation, nitrogen starvation and amino acid starvation all resulted in tRNA cleavage but produced distinct patterns of tRNA-halves in *S*.*cerevisiae*. The findings indicated that cleavage into tRNA halves is governed by complicated rules. They also suggested the possibility that the produced tRNA-half signal could help decode cell states. Work with human cell lines and mouse tissues revealed similar signals (1).

As it turned out, the responsible agent is a polypeptide that was originally discovered in 1985 during a search for angiogenic factors (17). The enzyme, dubbed Angiogenin, was later shown to be a homolog of pancreatic ribonuclease A that could target tRNAs (18,19) and cleave them at the anticodon loop (1). Angiogenin has since been linked to tRNA cleavage in a variety of settings (20–25). Angiogenin-related tRF production was also linked to immune responses following arsenite exposure, further implicating environmental factors in the regulation of tRFs (26).

Before continuing, it is worth mentioning a recent series of experiments by Dutta and colleagues that put into question the centrality of Angiogenin as the endonuclease that produces all tRNA halves. Specifically, they showed that while over-expression of Angiogenin increases the abundance of tRNA halves, this increase appears to be selective. Indeed, only 5′-halves and 3′-halves from tRNA^Glu^, tRNA^Gly^, tRNA^Asp^, tRNA^Val^, tRNA^Lys^, tRNA^Ser^, tRNA^iMet^ and tRNA^SeC^ responded significantly in those experiments (27). More intriguing was the finding that upon CRISPR-induced knockout of Angiogenin, the tRNA halves from tRNA^Asp^ and tRNA^His^ were the only stress-induced halves that exhibited dependence on Angiogenin. The experiments suggest that although Angiogenin is responsible for producing some tRNA halves, other currently unknown endonucleases must also be involved in the biogenesis of tRNA halves.

### tRNA fragmentation in the absence of stress

Parallel work also provided evidence that stress is not the sole mediator of tRNA cleavage. In fact, hormone signaling was shown to induce angiogenin-mediated tRNA cleavage in human prostate and breast tumor cell lines (28). In *S. cerevisiae*, Rny1p, a member of the RNase T2 family, was shown to promote cleavage in the anticodon loop (29). More recently, in *A*.*thaliana*, RNase T2 was linked to the production of tRNA halves, as well as of short tRFs through cleavage in the D and T loops (30). Additionally, Kay and colleagues showed that the RNase Z/ELAC2 produces 3′ trailer tRFs in human cell lines (31). With regard to localization, the presence of ELAC2 in the cytoplasm and the production of 3′ trailer tRFs in the cytoplasm is supported by the work of Dutta and colleagues (2) and Oh and colleagues (32). However, it is not clear how pre-tRNA molecules are exported to the cytoplasm. Notably, Rossmanith showed that ELAC2 localizes to the nuclei and mitochondria of T-Rex-293 cells (33), suggesting the possibility that ELAC2-derived 3′ trailer tRFs may not originate in the cytoplasm but rather be exported to it. We discuss the role of RNase Z/ELAC2 again below. The similarity of findings across the three life domains suggests that functional tRNA cleavage is conserved. Finally, it is worth noting that production of tRFs has also been linked to disease-causing mutations. For example, CLP1 founder mutations (34) were shown to dysregulate splicing of premature tRNAs (35). This in turn leads to the accumulation of tRFs from the 5′ end of precursor tRNAs (36).

## DEFINING TRFS

### Nomenclatures and categories of tRFs

In the tRF literature, one frequently finds the same tRF type under different names in different publications. tRNA halves are one characteristic example. Originally, tRNA halves were believed to be produced under cellular stress and were called ‘tRNA-derived stress-induced RNA’ or tiRNA (23,24). Subsequent analyses of NGS data showed that tRNA halves (28) as well as shorter tRFs (37) are also produced constitutively. In the case of tRNA halves, there is a further complication because the ‘tiRNA’ label was also introduced at around the same time by Mattick and colleagues to refer to ‘transcription initiation RNAs.’ The latter are short RNAs that arise from the same strand as the gene's transcription start site (38,39) and are preferentially associated with G+C-rich promoters. Transcription initiation RNAs are not related to tRNAs. Other names that have been used in the literature to refer to tRNA-derived fragments include tsRNAs for ‘tRNA-derived small RNAs’ (40) and tDRs for ‘tRNA-derived RNAs’ (41). The two public repositories of mammalian fragments, tRFdb (42) and MINTbase (43,44), adopted and have been using the abbreviation ‘tRF’ to refer to these fragments, in deference to the first time the term tRF was coined (2).

**Figure 1.**
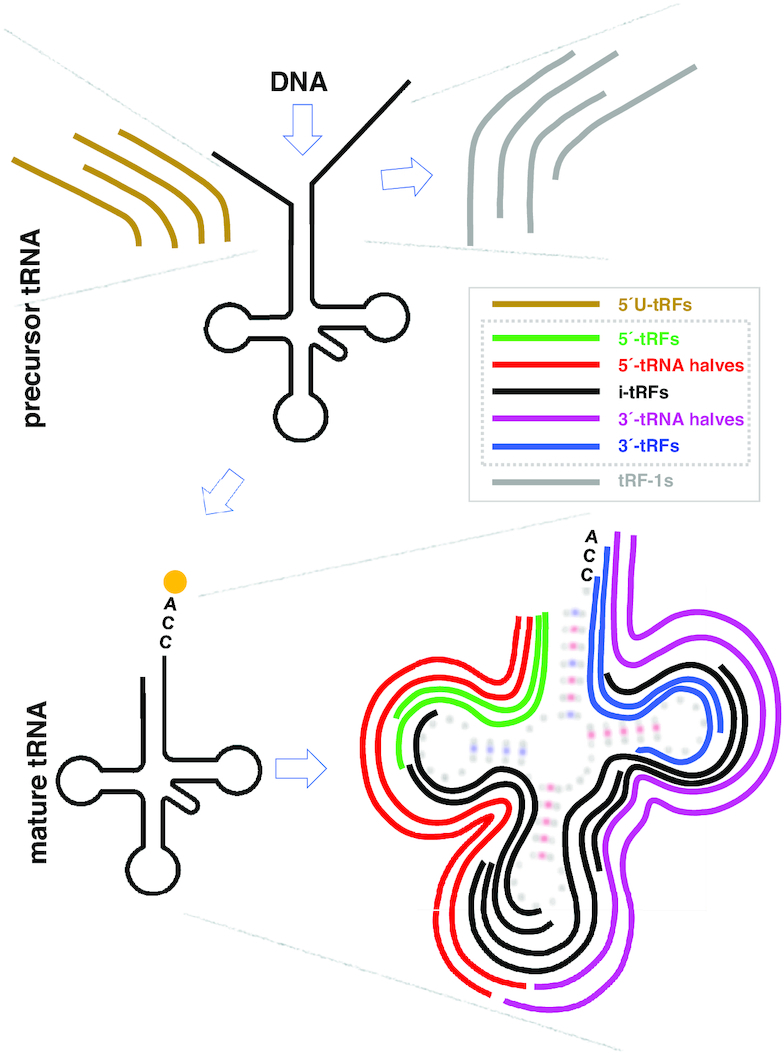
The structural types of the various tRFs. tRFs can be produced from either the precursor tRNA or from the mature tRNA. For more detail, refer to the description in the text.

A popular classification of tRFs is based on their position in relation to the parental tRNA sequence. Using this scheme, seven distinct categories can be identified (Figure 1). Five of these categories overlap the span of the mature tRNA and include: (i) 5′-tRNA halves or 5′-tRHs; (ii) 5′-tRFs or tRF-5; (iii) 3′-tRNA halves or 3′-tRHs; (iv) 3′-tRFs or tRF-3 and (v) i-tRFs, or internal tRFs. i-tRFs are wholly contained within the span of the mature tRNA, were initially discovered by analyzing NGS data (37), and are the most populous sub-category of tRFs (4,43). The remaining two categories include tRFs that overlap the precursor tRNA: (vi) 5′U-tRFs, which includes tRFs that comprise part of the 5′ leader sequence (45); and, (vii) tRF-1, which includes tRFs that comprise part of the 3′ trailer sequence (2). For uniformity of reference across the seven categories, we propose the following seven labels: 5′U-tRFs, 5′-tRFs, 5′-tRHs, i-tRFs, 3′-tRHs, 3′-tRFs and tRF-1.

Recent work reported a new category of tRFs, the sex-hormone-dependent tRNA-derived small RNAs, or SHOT-RNAs (28). Structurally, they are similar to 5′-tRHs. However, SHOT-RNAs are an entirely new category of molecules because of an important characteristic: the 3′ terminus of the 5′ SHOT-RNAs contains a cyclic-phosphate (cP) instead of a hydroxyl (OH) group. The 3′ terminus of 3′ SHOT-RNAs contains an amino acid. The presence of a 3′ cP makes SHOT-RNAs ‘invisible’ to standard NGS approaches. Interestingly, the production of SHOT-RNAs is sex-hormone dependent.

On a related note, it is important to point out that, in addition to remaining relatively expensive, NGS methods have some limitations when it comes to studying tRFs, even when the sought molecules do not have any 5′ or 3′ modifications. Internal tRNA base modifications could hinder the reverse transcription step during sequencing and result in the undercounting of bona fide tRFs, and new methods are being proposed for tackling this complication (46,47). These modifications were also thought to result in artificial 5′ endpoints for some tRFs or misread nucleotide(s) at the corresponding modified location(s). However, a recent large scale analysis of 11 198 TCGA datasets (4) did not find evidence that the presence of these modifications results in artificial 5′ termini or that the modifications distort the identity of the tRFs that can be mined from NGS datasets.

### Labeling individual tRFs

In addition to the need for a universal moniker for these fragments, there is also a need for a methodology by which one can refer to a specific tRF. In fact, there is currently no standardized nomenclature for tRFs. This is unlike the class of microRNAs (miRNAs), where a recent multi-team effort recognized a similar need for standardization and proposed a universal scheme for labeling miRNA isoforms (48,49), which we discuss below.

In the case of tRFs, the problem is compounded by the existence of multiple isodecoders for each isoacceptor. These isodecoders can share sequence segments, or even their entire sequence, while appearing at different chromosomal locations. There are at least three generic approaches by which one can refer to a specific tRF: (1) arbitrary numbering; (2) tRNA-label driven; and, (3) nucleotide-sequence driven. The first of the three is similar to what is being used by miRBase (50) to label microRNAs. The approach requires a dedicated team that issues and maintains labels, and will not be discussed here.

The second, tRNA-label driven approach builds on the label of the tRF’s parental tRNA isodecoder (e.g. tRNA-Arg-CCT-1) by adding the starting and ending positions of the tRF to the isodecoder's label. This is the approach taken by ‘tDRnamer’ (http://trna.ucsc.edu/tDRnamer/docs/). In those cases where the same nucleotide sequence appears in multiple isodecoders of the same isoacceptor, one needs to keep track of that possibility by further augmenting the tRF's label. While simple to execute, this approach gives rise to a conundrum. First, it assumes that the tRNA space is static with regard to the number of isodecoders, whose labels form part of the tRF label, for a given isoacceptor. The number of isodecoders can increase or decrease as new genomic assemblies become available and genomic regions get added or deleted. Second, the naming scheme results in the same tRF having multiple labels. For example, the 22 nt tRF with sequence GGGGGTGTAGCTCAGTGGTAGA, is present in five isodecoders of tRNA^AlaAGC^, one isodecoder of tRNA^AlaCGC^, five isodecoders of tRNA^AlaTGC^, one isodecoder of tRNA^CysGCA^ and one isodecoder of tRNA^ValAAC^. Also, in different organisms, the same tRNA-isodecoder-label-and-tRF-interval combination could refer to different nucleotide sequences, thus necessitating the use of the organism's name to ensure disambiguation of the nucleotide sequence.

The third, sequence-driven approach takes into account the actual nucleotide sequence of the fragment. We previously proposed this scheme as a possible solution to the tRNA-label conundrum mentioned above. The scheme uses a universal ‘license plate’ to label each tRF. The label (‘license plate’) is derived from the tRF sequence itself and is based on the digits 0 through 9 and the 22 upper case letters of the English alphabet that remain after excluding A, C, G and T (43,44). For example, the 18 nt i-tRF CGCCTGTCACGCGGGAGA is present in 13 distinct isodecoders of tRNA^AspGTC^ that are located in *eight* different chromosomes. To refer to this tRF, one simply uses its license plate, tRF-18-L7S5QKX. The license plate comprises three parts: the prefix ‘tRF,’ the infix ‘18’ that denotes the length of the short RNA, and the suffix ‘L7S5QKX’ that encodes the nucleotide sequence. The mapping between a tRF and its license plate is one-to-one. Consequently, one can recover the correct tRF sequence from a given license plate. In addition to publishing the rules for generating license plates and converting them back to nucleotide sequences, we also made codes and an interactive interface freely available for this purpose (https://cm.jefferson.edu/LicensePlates/). While the scheme is not perfect, it offers several benefits: because it is sequence-based, there is a 1:1 correspondence between a tRF and its license plate; it is model organism-agnostic; and, license plates can be generated by anyone without requiring the intervention of a brokering entity. In other words, this labeling system does not depend on the availability of funding to sustain a team that issues and maintains these labels. It is important to also note that a key property of the license plate is that it is ‘sticky:’ it exists independently of changes to the genome assembly, genome assemblies for different strains of the same species, or different species of the same genus. Naturally, a single license plate can be used to refer to a tRF that is evolutionarily conserved. License plates are being used by MINTbase (43,44).

Lastly, it is worth pointing out that the license-plate labeling scheme can be extended to other types of short RNAs. For example, it was adopted by the recently-proposed standard for labeling, reporting, and comparing miRNA isoforms, which are known as isomiRs (48). As with the tRFs, the rules remain the same with the exception of the prefix, which now is ‘iso-’ (instead of ‘tRF-’). More recently, we extended it to ribosomal RNA-derived fragments, which are also known as rRFs (51). The rules for generating license plates for rRFs remain the same with the exception of the prefix which is now ‘rRF-’.

### Genomic idiosyncrasies that affect the identification of tRFs

Early studies of tRFs relied on a simple approach whereby sequenced reads were mapped on the known isodecoder sequences using standard mapping software. This was essentially the process that had been used to analyze miRNAs for many years. While reasonable, this approach does not take into account the fact that the same nucleotide sequence may appear in different isodecoders of the same isoacceptor, or even different isoacceptors (see previous section). And it does not account for three more observations that complicate the task further.

First, a study of the RepeatMasker (A.F.A. Smit, R. Hubley & P. Green RepeatMasker at http://repeatmasker.org) output for the human genome reveals that the latter contains hundreds of instances of incomplete tRNAs (52). The reader is referred to a characteristic example relating to the 5′ tRNA halves of tRNA^IleTAT^ (52).

The second complication relates to the discovery of several hundred regions in the human genome that closely resemble known nuclear and mitochondrial tRNAs: we dubbed these regions ‘tRNA lookalikes’ (53). The lookalikes co-localize with the known *bona fide* tRNAs (*P*-val < 4.0E−03) and several show evidence of cell-type specific transcription. Most interesting is the fact that ∼70% of these lookalikes closely resemble *mitochondrial* tRNAs. When we investigated the mitochondrial connection further, we found that many animal genomes, from human to marsupials, harbor between several dozen to hundreds of copies of their mitochondrial tRNAs in their nuclear genomes. This is also true for at least one plant, *A. thaliana* (Rigoutsos, unpublished). The persistence of the phenomenon and the cell-type specific expression of the human tRNA-lookalikes suggests that they have functional roles, especially given that mitochondrial tRNA are known to produce numerous tRFs (4,37,43,44,54–57). While the purpose of the lookalikes is not currently understood (58) their genomic copies need to be taken into account when seeking to identify tRFs in NGS data.

The third complication has to do with the fact that many short nucleotide sequences can exist at multiple genomic locations (59), both protein-coding and non-coding (60). Consequently, each candidate tRF sequence needs to be examined against the rest of the genome to determine whether it is also present at a non-tRNA locus. For example, the 23 nucleotide tRF TGGTGGTTCAGTGGTAGAATTCT is present in 13 isodecoders: eight isodecoders coding for tRNA^GlyGCC^, 3 isodecoders coding for tRNA^GlyCCC^, and one isodecoder coding for each of tRNA^GluTTC^ and tRNA^ValCAC^. However, this sequence is not unique to tRNA space, and thus not necessarily a tRF: it matches exactly five partial tRNAs as well as one unannotated region that is present only in primates.

Therefore, it is important to take the entire genome into account when determining the genomic source of a candidate tRF. We stress that longer tRFs such as the tRNA halves are not immune from this consideration. Of the 375 tRNA halves that show evidence of transcription in human tissues (43), 66 (17.6%) have genomic instances outside of the known tRNAs. And of the 6375 tRFs that show evidence of transcription and have lengths between 25 and 30 nt inclusive, 1514 (23.7%) are not unique to tRNA space either (43).

These interconnections complicate the labeling of tRFs, the unambiguous identification of a candidate tRF’s parental tRNA, and the identification of tRFs in NGS data (37,52). In the ‘Tools and Databases’ section below, we discuss how different mapping solutions approach these problems.

## ON THE BIOGENESIS AND FUNCTION OF tRNA FRAGMENTS

**Figure 2.**
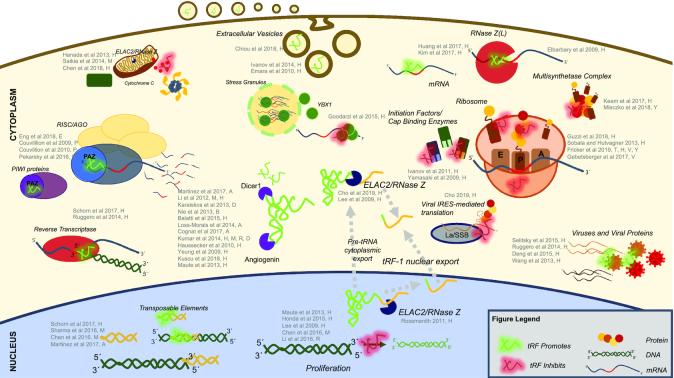
Visual summary of tRF mechanisms in the literature during the last 10 years. These illustrations represent tRF mechanisms and behaviors that involve either direct or indirect effects on protein or mRNA and have been confirmed with experimentation. Citations are provided, including first author last name and year of publication. Note the use of single letter codes to denote the organism in which the work was done: A – *A. thaliana*; B – *B. mori*; D – *D. melanogaster*; E *– A. aegypti*; H – *H. sapiens*; M – *M. musculus*; P – *T. thermophilus; R – R*. norvegicus; T *– T. brucei* or *T. cruzi*; V – *H. volcanii*; and, Y – *S. cerevisiae*. Red shading indicates processes that are downregulated by tRFs whereas green shading indicates activities that are upregulated by tRFs.

Figure 2 provides a visual summary of findings to date from what is an active area of research. We discuss individual findings in more detail in the sub-sections that follow.

### Enzymes responsible for cleaving tRNAs into fragments

Notwithstanding the tRNA halves, the other tRF types are typically shorter with sizes reminiscent of miRNAs. This length similarity raised the possibility that tRF production is Dicer-dependent. This has now been investigated in several organisms.

Early evidence suggested that the tRF production in *S. pombe* is Dicer-independent (61). Subsequent work with *P. infestans* showed that knockdown of Argonaute affected tRF levels but knockdown of Dicer did not (62). On the other hand, work in mammalian cells demonstrated that at least some tRFs are produced in a Dicer-dependent manner (63–65). A subsequent systematic meta-analysis of available data showed that Dicer is required for the production of some tRFs in human cell lines, but not in *M. musculus*, *D. melanogaster*, or *S. pombe* (66).

At present, tRF production is known to depend on several enzymes including Angiogenin, RNase T2, Dicer and RNase Z/ELAC2. The recent finding that only some tRNA halves depend on Angiogenin (27) suggests the existence of yet-to-be-defined cleavage mechanisms. Additionally, as we discussed above, multiple types of stress and hormone signaling may also be involved.

Other proteins have also been investigated, providing further insights into the regulation of tRNA cleavage. Specifically, activity of the cytosine-5 methyltransferase DNMT2 was found to prevent the cleavage of stress-induced tRNA halves in *D. melanogaster* (67). On the other hand, loss of cytosine-5 methylation in mice and humans leads to increased production of Angiogenin-dependent 5′ tRFs through a mechanism that involves lack of methylation at key sites of the mature tRNA structure (68). Related to this observation, it was shown recently that ALKBH3 activity results in removal of m^1^A and m^3^C tRNA modifications, resulting in sensitivity to angiogenin cleavage (69). DNMT2 was also shown to be essential for the production of methylated tRFs in mouse sperm (70).

Beyond these enzymes, alternate partners responsible for cleavage and processing of tRFs remain unknown. Recently, Polacek and colleagues described the function of a 3′ tRNA half from tRNA^Thr^ in *T. brucei* that post-transcriptionally regulates translation via ribosome interactions (71). These authors note that *T. brucei* lacks Angiogenin and Rny1p homologues, and that the observed tRNA halves do not depend on Dicer, further emphasizing that currently unknown proteins are linked to tRF biogenesis.

### tRNA fragments as functional short RNAs

Scattered reports on disease-associated tRFs or degradation products appeared throughout the second half of the 20th century (72–75). For a description of how the thinking in this field evolved, the various roles of Angiogenin, and reports on tRFs in different settings, we refer the reader to the excellent review by Paul Schimmel (76).

Initially, tRFs were not mechanistically evaluated as individual causative agents of cellular behavior or disease. This changed when the study of miRNAs and small interfering RNAs (siRNAs) allowed the role of short non-coding RNAs in the cell to be fully appreciated. Individual reports on tRFs appear in the public record through the late 1990s, but these reports discuss fragments mostly as experimental tools.

In 2001, Bartel *et al.* reported results of an extensive effort to discover and name new miRNAs and siRNAs in *C. elegans* (77), whose genome had been published three years earlier. The availability of an assembled genome allowed the researchers to screen all non-coding RNAs in an unbiased approach, and to identify and quantify RNAs from any region of the annotated genome. While they noted that some Dicer-processed RNAs mapped to tRNA genes, they did not further characterize them at the time.

Dutta and colleagues were first to characterize all short non-coding RNAs in a set of human prostate cancer cell lines using ‘454 deep sequencing’ (2). While a large number of reads mapped to miRNAs, a considerable percentage mapped to annotated tRNAs, in accordance with the observation made years earlier in *C. elegans* by Bartel *et al.* Dutta's team validated the expression of these tRNA-mapped reads using northern blots and splinted ligation assays, and concluded that these were products of targeted cleavage, similar to tRNA halves. One abundant tRF in particular, dubbed ‘tRF-1001,’ was shown to be required for HCT116 proliferation and cell cycle progression. tRF-1001 belongs to the tRF-1 category (see above) and was found to be produced during tRNA maturation by RNase Z/ELAC2 cleavage of 3′ tRNA trailers.

## EVIDENCE FOR THE MULTI-FACETED ROLES OF TRNA FRAGMENTS

### Function of tRFs: tRFs and the Argonaute/Piwi proteins

The members of the Argonaute family of proteins are characterized by the presence of the PAZ and PIWI domains, and are highly conserved (78). The family comprises the Argonaute sub-family and the Piwi subfamily. The Argonaute sub-family members are ubiquitous and were originally reported to interact with miRNAs (79). The Piwi subfamily members have been reported only in the germline and interact with piRNAs (80). Organisms typically encode multiple Argonaute (AGO) and PIWI proteins.

The size similarity between most tRFs and miRNAs/siRNAs led researchers to hypothesize that tRFs behave like miRNAs and influence protein abundance in an Argonaute-dependent manner. Several early studies showed the association of a number of tRFs with all four Argonaute proteins, indicating that some tRFs enter the RNA interference (RNAi) pathway (31,81). In fact, a 3′-tRF from tRNA^GlyGCC^ was shown to promote the degradation of RPA1 to arrest B cell lymphoma proliferation, in a sequence- and Argonaute-dependent manner (65). In *A. thaliana*, tRFs were found enriched in various Argonaute immunoprecipitation (IP) experiments (82). Follow-up work further characterized the interactions between tRFs and AGO in *A. thaliana*, and described tRF populations that were specific to sub-cellular compartments (83). In *A. thaliana*, it was shown that some tRFs load directly onto AGO1 after processing by the Dicer homologue DCL1 (84). Importantly, this interaction was shown to promote degradation of RNAs produced from active transposable elements (TE) targeted by these tRFs. AGO1 and AGO2 tRF loading has also been observed in *D. melanogaster* (85) and in *Bombyx mori* (86), at comparable levels to that observed in a large meta-analysis across species (66). For mammalian cells, Dutta and colleagues showed that tRFs are loaded on Argonaute (66) whereas Rigoutsos and colleagues showed that the Argonaute-loading of tRFs is cell-type dependent (37).

Mammalian tRFs have been studied in greater depth than non-mammalian tRFs, and the evidence for miRNA-like activity extends to a mechanistic level in this context. For example, two previously annotated miRNAs were reclassified as tRFs and shown to interact with AGO2 promoting proliferation arrest in a Chronic Lymphocytic Leukemia (CLL) model (87). Endogenous tRFs were shown to load on AGO1 and AGO2 in mouse embryonic stem cells and HEK293 cells, and to promote cleavage of sequence-matched artificial RNA targets (88). Recent reporting provided more evidence in support of tRF loading on Argonaute proteins whereby they direct the degradation of sequence matched targets (89). Importantly, this last study re-affirms Dicer independence for the studied tRFs and implicates yet more proteins in the RNA-induced silencing complex as tRF binding partners.

As with AGO, tRFs have been observed to interact with PIWI proteins as well, in a variety of contexts. Croce and colleagues demonstrated that two tRFs interact with PIWIL2 in HEK293T cells (40), in a mechanism that they hypothesize may promote targeted methylation of DNA via tRF sequence recognition. Other reports implicated PIWI proteins in tRF-binding interactions in human somatic cells (90), *B. mori* (91) and *T. thermophila* (92,93). The *B. mori* studies showed that the 5′-tRNA halves from tRNA^AspGTC^ and tRNA^HisGTG^ serve as the precursors for the shorter 5′-tRFs that in turn interact with PIWI. In fact, the production of 5′-tRFs from tRNA^HisGTG^ was shown to depend on TH1GL (94,95). The *T. thermophila* studies demonstrated that only 18–22 nt 3′-tRFs in this species constitutively interact with a catalytically-inactive PIWI protein Twi12. The findings also suggested that the specificity of tRF interactions with Twi12 may be indicative of a coordinated tRNA degradation pathway. Alternative results demonstrate that differences in human tRNA isodecoder gene expression correlate with differences in the abundance of immature tRNAs and tRFs, but not the abundance of the mature tRNAs themselves within a range of human tissues (96). It is currently an open question whether tRF interactions with PIWI-related proteins guide target interactions or are a result of degradation pathways across species.

### Function: tRFs and other RNA-binding proteins

Beyond the Argonaute family, tRFs are known to interact with several other RNA binding proteins (RBP). YBX1 is an RBP that stabilizes mRNAs in the cytoplasm prior to translation. In breast cancer cells, several i-tRFs were shown to compete with the 3′-untranslated regions (3′-UTRs) of oncogenes for binding to YBX1. By displacing YBX1 from these oncogenes, tRFs could arrest tumor progression in human breast cancer cell lines (97). More recent work expanded on this concept of competitive displacement. Falconi *et al.* reported that in breast cancer cells an i-tRF from tRNA^Glu^ can counter nucleolin's repression of p53, leading to the p53 translation and tumor suppression (98).

tRFs are also known to interact with reverse transcriptase (RT). Specifically, in mouse embryonic stem cells and in transformed cells, abundant 18 nt 3′-tRFs containing -CCA tail tRNA modifications were shown to target the primer binding site (PBS) of LTR retrotransposons in a sequence-dependent manner. The PBS is essential for reverse transcription of endogenous retroviruses (ERVs). By competing for binding the PBS with the RT, the tRFs effectively blocked reverse transcription (99). The authors also observed that 22 nt tRFs are able to silence coding-competent ERV through RNAi.

Hatzoglou and colleagues studied the apoptotic machinery (21) and found that Angiogenin suppresses formation of the apoptosome and promotes accumulation of tRNA halves in mouse embryonic fibroblasts. After isolating cytochrome C-ribonucleoprotein complexes from these fibroblasts, they found stress-induced tRNA halves to be the major interacting RNAs. They also showed that apoptosis of mouse cortical neurons due to hyperosmotic stress could be arrested by Angiogenin overexpression, presumably through a direct interaction between cytochrome C and induced tRNA halves. More recently, tRNA halves were independently shown to associate with cytochrome C in HeLa cells (69).

### Function: tRFs and intergenerational inheritance

The connection between diet and tRNA halves in serum was reported early on (100). A more recent study demonstrated that low protein diet (LPD) in mice increased the number of recoverable small RNAs in sperm (101). Specifically, it was found that abundant 5′-tRFs from tRNA^Gly^ are introduced to sperm by epididymal epithelial cells, as the sperm transits the epididymis and encounters epididymosomes. These tRFs were shown to associate with MERVL TEs in embryonic stem cells and in the developing embryo, potentially influencing the observed low birth weight of pups from parents on LPD.

A second study investigated the effects of high fat diet (HFD) on tRF behavior. It was found that 5′-tRNA halves in sperm are both enriched and differentially methylated in mice on HFD (102). The authors observed metabolic pathway deficits leading to glucose intolerance in offspring from zygotes injected with these HFD enriched tRFs, without a related increase in DNA methylation at CpG islands. They concluded that tRFs mediate ‘intergenerational inheritance’ through an unknown mechanism. A third study similarly reported the activity of mouse sperm tRFs in the context of intergenerational inheritance of glucose and lipid metabolism deficits (103).

More recently, it was demonstrated that the methyltransferase DNMT2 is necessary for the intact production of hypermethylated and hyperabundant tRFs, 30–40 nt long, in mouse sperm (70). Hypermethylated instances of these tRFs, but not un-methylated or hypo-methylated ones, lead to the intergenerational transmission of paternally acquired metabolic disorders through currently elusive mechanisms.

### Function: tRFs and the stability of RNAs

Other proteins have been implicated in tRF-mediated control of mRNA stability. In a series of papers, Elbarbary and colleagues showed that tRNase Z(L), the endonuclease responsible for tRNA 3′ end maturity, can interact with either miRNAs or tRF species to direct cleavage of a sequence-matched RNA (104,105). Specifically, both *in vitro* and *in vivo* interactions between a 5′-tRNA half from tRNA^Glu^ and the PPM1F mRNA promoted degradation of the mRNA target.

Interactions between sequence-matched mRNA targets and tRFs in the absence of a protein mediator have also been characterized. A 17 nt tRF from tRNA^Leu^ (formerly annotated as miR-1280) was shown to interact directly with the Notch ligand JAG2 (106). This interaction prevented translation of this critical Notch1 and Notch2 receptor connector, preventing expansion and behavior of stem cell-like cells in HCT116 and HCT15 colorectal cancer cell lines. This interaction appears to be critical in preventing metastatic behavior of HCT116, through regulation of factors normally promoting the ‘pro-metastatic niche’.

Another study showed that a tRF from tRNA^LeuCAG^ stabilizes the RPS28 and RPS15 mRNAs during translation, thereby increasing the abundance of these ribosomal proteins. When this tRF is inhibited in HeLa cells, stability of the 40S ribosomal subunit is compromised due to loss of these proteins followed by apoptosis (107).

Interestingly, tRFs have also been implicated in the stability of other noncoding RNAs. Rando and colleagues recently commented on a mechanism whereby a 5′-tRF from tRNA^GlyGCC^ interacts with hnRNPF and hnRNPH to influence the stability of Cajal bodies and the activity of the U7 snRNA (108). This finding implicates tRFs in a global mechanism that regulates the production of noncoding RNAs. Future work should test the hypothesis that tRFs can act as master regulators.

### Function: tRFs and the ribosome

The interactions of tRFs with the ribosome are well characterized. In several studies, Anderson and colleagues investigated the mechanism of stress-induced (109) Angiogenin-cleaved tRNA halves (110) and showed that some tRNA halves inhibited protein translation by directly interacting with the active eukaryotic translation initiation factor—phospho-eIF2α—in the actively translating ribosome (110). At the same time, tRNA halves were further implicated in stress granule (SG) assembly in U2OS cells, as part of a coordinated program of cellular behavior, presumably activated to sequester mRNA and prevent translation during times of nutrient scarcity (24). It was subsequently demonstrated that activity of tRNA halves from tRNA^Cys^ and tRNA^Ala^ under stress conditions inhibited protein synthesis. In this context, tRNA halves work by directly preventing eIF4G/eIF4A from stabilizing capped mRNAs, thereby preventing the ribosome from recognizing and initiating translation of these RNAs (23). This is achieved by a poly(G) motif that is present in the 5′ tRNA halves from tRNA^Cys^ and tRNA^Ala^ and promotes formation of a G-quadruplex structure (111).

Interestingly, these authors reported that interaction between YBX1 and these stress-induced tRNA halves results in SG formation, but not in translation inhibition. The cumulative interpretation of these findings presents an interesting model for the behavior of specific tRFs. Under stress conditions, angiogenin cleaves tRNAs to promote tRNA half formation. Those tRNA halves with stability-conferring poly(G) motifs assemble into G quadruplex structures that in turn confer function. Other tRNA halves, which accumulate due to the same initiating angiogenin activity, interact with YBX1 instead. Through both mechanisms, downstream pathways are activated in order to slow cell growth and promote recovery from stress.

Hutvagner and colleagues have been studying a different aspect of the interactions of tRFs and the ribosome. After characterizing the Dicer-dependent production of tRFs (64), they followed up by demonstrating that 5′-tRFs from tRNA^Gln^ are also capable of arresting translation *in vitro* through interaction with active polysomes (112). Their findings present a distinct mechanism whereby tRFs inhibit translation. Nonetheless, the findings agree with contemporaneous work that showed that a 5′-tRF from tRNA^Val^ binds to the small ribosomal subunit of *H. volcanii*, competes with mRNA binding and attenuates translation (113).

In follow-up work, Hutvagner and colleagues returned to the question of tRFs interfering with ribosomes and reported that 5′-tRFs from tRNA^Gln^ interact with the Multisynthetase Complex (MSC). The MSC is a cytoplasmic focus of several aminoacyl-tRNA synthetases and ribosomal components that coordinates assembly of mature ribosomes. They showed that direct binding of the MSC by this small tRF in HeLa cells disrupts MSC stability, inhibits maturation of ribosomes, and globally represses translation (114). A similar finding was subsequently reported in *S. cerevisiae* (115).

As with miRNAs, tRFs have been described in bidirectional control relationships with ribosome function. The majority of the evidence suggests that 5′-tRNA halves and other tRFs inhibit the function of ribosomes, in order to promote stress responses. However, a recent report described how in nutrient-deprived *T. brucei* a CCA-lacking 3′-tRNA half from tRNA^Thr^, which is cleaved by an unknown mechanism, associates with the large subunit of active ribosomes (71). This 3′-tRNA half does not promote formation of stress granules. Instead, because of its length, it can prime large ribosomal subunits and promote translation by increasing the affinity of ribosomes for mRNAs destined to be translated after relief of stress. These findings collectively point towards a mechanism for tRF action in *T. brucei* whereby 3′-tRNA halves promote ribosomal function in a length-dependent manner. This is unlike 5′-tRNA halves that inhibit ribosomal function, in a sequence- or modification-dependent manner. The authors provided evidence of the generality of this observation by demonstrating that this 3′-tRNA half also stimulates translation in *H. volcanii*, *S. cerevisiae* and HeLa extract in *in vitro* assays, pointing to similar potential functions in other organisms.

Through a variety of discrete mechanisms and in several different species, tRFs appear to join their size-matched miRNA/siRNA cousins in inhibiting translation of mRNA targets. However, while miRNA/siRNA translational inhibition is sequence-specific by the nature of target-seed interactions, translational arrest promoted by tRFs is not at all target specific, but global in nature and due to interaction with complexes that support translation, such as small ribosomal subunits and tRNA synthetase complexes.

## tRNA FRAGMENTS IN DISEASE SETTINGS

### Disease: tRFs and stemness

One exciting discovery of a direct mechanism of tRF involvement in disease involves bone marrow stem cell differentiation (116). Specifically, Bellodi and colleagues showed that pseudouridylation of tRFs modulates protein biosynthesis. During self-renewal of human embryonic stem cells (hESC), the pseudouridylase PUS7 recognizes a motif present in several mature tRNAs—UGUAG—and pseudouridylates the central uridine. Pseudouridylated bases represent a handful of the hundreds of characterized modifications on mature tRNA (117). tRFs bearing both this pseudouridine and the 5′ poly(G) runs, initially characterized by Ivanov and Anderson, selectively associate with the translation initiation factor PABPC1. On the other hand, non-pseudouridylated tRFs interact with YBX1 and other RBPs. Knockdown of PUS7 or loss of pseudouridylated tRFs prevents an arrest in translation normally required to slow the growth of hESCs long enough for them to differentiate into myeloid precursors. Surprisingly, PUS7 is encoded in a region known to be deleted in myelodysplastic syndromes (MDS)—rare disorders of blood cell progenitor regulation that cause diverse symptoms and a high risk of leukemic transformation. MDS are associated with deletions or complete monosomy of chromosome 7, in a currently unknown mechanism. The findings present compelling evidence that dysregulation of a tRF-based regulatory mechanism may underlie these disorders.

Other roles for tRFs in stemness have also been discussed. tRNA methylation by NSun2 has been linked to stress-induced reduction in stem cell protein synthesis. Frye and colleagues showed that loss of NSun2 results in accumulation of 5′-tRNA halves in mouse tumor stem-cell models (118). Notably, increased abundance of 5′-tRNA halves promoted tumor activity through translation of stress response, migration, and adhesion proteins. Further, this work suggests that NSun2 knockout allows squamous tumors to escape sensitivity to stress mediated by 5-fluorouracil (5FU) for example, and that this escape mechanism potentially depends on 5′-tRNA half production. This finding has implications for increasing chemotherapy efficacy through the addition of tRNA methylation inhibitors to regimens involving 5FU.

### Disease: tRFs and neurologic disorders

Several links between tRF behavior and neurologic disorders have been introduced in the recent literature. Ivanov and Anderson reported that the Angiogenin-associated G-quadruplex structures also promote motor neuron recovery under apoptotic conditions, revealing a potential mechanism for Amyotrophic Lateral Sclerosis (ALS) (111). These authors demonstrated that the C9orf72 ALS-associated pathogenic RNA repeats directly interfere with this protective behavior of tRNA halves. Both ALS and Parkinson's Disease (PD) are associated with mutations in the angiogenin gene (119–121). Recently, we showed that a small panel of serum tRFs may distinguish PD patients from controls with high sensitivity and specificity (55). And tRNA halves and Angiogenin have been investigated for their protective role in a PD experimental model (122,123). However, the relationship between Angiogenin and neurodegeneration may be more complicated (124). In other work, Li and colleagues demonstrated that tRFs are involved in a response to ischemia in rat brain, and specifically inhibit proliferation of human umbilical vein cells (HUVEC) *in vitro* (125). Finally, study of mutations of CLP1 (see above) revealed that sensitization of cells to p53-mediated apoptosis by a 5′U-tRF underlies a familial motor neuron disorder (36).

### Disease: tRFs and viral infections

tRFs have also been investigated as components of human responses to viral infection. Two studies showed that 5′-tRFs produced from tRNA^Glu^ in response to Angiogenin signaling after infection by the respiratory syncytial virus directly promote infection (126,127). Infection is specifically mediated through ‘trans-silencing’ involving interactions between the 3′-UTR of the antiviral APOER2 receptor and the 3′ ends of these tRFs. Infection with the hepatitis viruses B and C was also shown to induce tRF expression in liver tissues (41). tRFs were also linked to infections by the human T-cell leukemia virus type 1 (HTLV-1) and shown to prime reverse transcriptase to promote HTLV-1 infection and potentially leukemic transformation (128). Recently, 3′-tRFs from tRNA^SerTGA^ were shown to interact with La/SSB nuclear-cytoplasmic shuttling proteins in the human hepatocellular carcinoma model Huh7 (32). Oh and colleagues describe a role in which these tRFs depend on La/SSB chaperones for stability and prevent RNA viruses like HCV from hijacking La/SSB for initiation of IRES-mediated translation.

Recently, tRFs were also shown to act as regulators of normal immune responses. In activated human T-cells, select 5′-tRFs and i-tRFs are secreted actively in extracellular vesicles (EVs), and their identities differ from those found in EVs secreted by resting T-cells (129). Inhibition of EV release leads to accumulation of these tRFs in multivesicular bodies (MVBs). Inhibition of these tRFs using antisense oligonucleotides promoted T-cell activation, pointing toward a potential role for tRFs in normally regulating the level of T-cell activation. Importantly, tRFs have been implicated in the pathogenesis of leukemia, underscoring the potential importance of tRFs as immune cell regulators (40,130).

### Disease: tRFs, cancer and overall survival

Among diseases, the links between tRFs and cancer were among the earliest explored. Specifically, silencing of a fragment from the tRF-1 category was shown to influence tumor cell line proliferation (2). For human tissues, Rigoutsos and colleagues published the first explorations of the links between tRFs and homeostasis or disease, and reported on the expression profiles of tRFs in lymphoblastoid cells derived from healthy individuals and in multiple subtypes of breast cancer (37,57). Since then, they have also discussed tRFs in prostate cancer (56), uveal melanoma (54), lung cancer (48), bladder cancer (48), and kidney cancer (48). In the case of uveal melanoma, they showed that tRFs are linked to overall survival (54). Parallel work investigated the expression and function of tRFs in: liver cancer (41), prostate cancer (45), ovarian cancer (131), colorectal cancer (106), hormone-responsive cancers (28), breast cancer (97), non-small cell lung cancer (132), B-cell lymphoma (65), and chronic lymphocytic leukemia (130). These many connections raise the possibility of using tRF expression as a proxy for disease status. We discuss this below.

### Disease: dependencies of tRF production on disease type/subtype, tissue type and state and personal attributes

A result that was enabled by the availability of large amounts of data was the reporting by Rigoutsos and colleagues that the identity and abundances of tRFs depend on cellular context. Specifically, they were first to show that these tRF attributes are modulated by the type of the tissue at hand, the state of the tissue (healthy or diseased) and the type/subtype of the disease (37,54–57). They have since extended these findings to 32 tissue-type/cancer-type combinations and showed that each such cancer context exhibits its own characteristic profile of tRF transcripts (4).

Additionally, they reported that the transcriptional profiles of tRFs also depend on personal attributes including sex (4,37,55), population of origin (37), and race/ethnicity (37,56,57). Importantly, they showed that these tRF dependencies are linked to concomitant putative regulatory differences between people who differ by one or more of these characteristics. So far, they have reported such findings in prostate cancer (56), triple negative breast cancer (57), lung cancer, bladder cancer and kidney cancer (4), and Parkinson's disease (55).

### Disease: systems-level analyses of tRF function and genome architecture

With a few exceptions, the biogenesis and functional spectrum of tRFs remains poorly characterized. While it is known that some tRFs are loaded on Argonaute (65,66,89) not all tRFs with lengths matching that of miRNAs do so (37,66). In other words, a miRNA-like length is not sufficient for Argonaute loading. Moreover, analysis of CLIP-seq data from three cancer model cell lines showed that such loading is cell-type specific (37).

**Figure 3.**
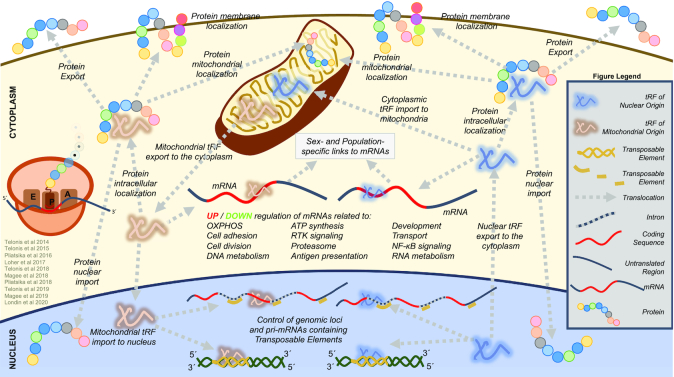
Visual summary of the findings that emerged from the analyses of all TCGA cancers previously described by Rigoutsos and colleagues (4,37,43,52–57,59). The analyses are based on computing positive and negative correlations between tRFs and mRNAs (‘co-expression networks’). These correlations capture direct molecular couplings as well as indirect interactions (e.g. decoying events and propagated regulatory effects). For those mRNAs that participate in the correlations with tRFs, their intronic and exonic lengths, their respective genomic spans, the repetitive content of those genomic spans, and the cellular localization of the proteins that are produced by these mRNAs were also examined. The picture that emerges is complex, yet remarkably consistent across the 32 TCGA cancer types. Summarily, numerous tRFs of nuclear and mitochondrial origin are correlated with mRNAs. These mRNAs belong to key processes including development, receptor tyrosine kinase signaling, the proteasome, and metabolic pathways. The mRNAs that participate in positive correlations are generally shorter and enriched in repetitive elements. On the other hand, the mRNAs that participate in negative correlations are longer and depleted in repetitive elements. Perhaps the most striking finding that emerges from this analysis is the prominent participation of the mitochondrial tRFs in correlations with mRNAs that belong to processes that are not mitochondria-specific. This raises the possibility of the participation of mitochondrial tRFs in an ‘information exchange’ that could be implemented in one of two ways: either the mitochondrial tRFs exit the mitochondrion and are shuttled to the cytoplasm and the nucleus, or they are produced by transcription of the mitochondrial ‘tRNA-lookalikes’ encoded in the nuclear genome and subsequent processing. The picture that emerges suggests that older and younger categories of repetitive elements as well as gene architecture are tightly-coupled with tRFs of nuclear and mitochondrial origin to a wider information exchange framework. The use of dashed gray lines in this figure is meant to show relationships that arise from the analysis of TCGA cancers and await independent experimental validation.

Rigoutsos and colleagues investigated tRF functions by tapping into TCGA datasets, computing correlations between tRFs and mRNAs, and examining the genomic and composition properties of these mRNAs and the localization of the produced proteins (4). Figure 3 summarizes the findings of this and related studies.

First, Rigoutsos and colleagues mined tRFs in 10 274 TCGA samples and retained 20 722 tRFs that exceed threshold in at least one sample. For only the tumor samples, they identified mRNAs that also exceeded threshold, computed positive and negative tRF:mRNA correlations, and kept only statistically significant ones. For each cancer type, they computed GO terms that were enriched among the tRF:mRNA correlations and focused on GO terms that recurred across the 32 cancer types. In many cancer types, tRFs were found to be associated with mRNAs from developmental processes, signaling, the proteasome, and metabolic pathways. These correlations likely reflect a mix of direct molecular interactions and indirect events. While the *same* GO terms were found enriched across cancers, the tRF:mRNA pairs giving rise to these enrichments *differed* from cancer to cancer.

These analyses also uncovered unexpected, wide-ranging relationships between the transcriptome and the genome. Specifically, relationships between tRFs and the genomic architecture of mRNAs with which the tRFs are correlated. In particular, tRFs were shown to be positively correlated with genes that have shorter exons and shorter introns and a higher density in repetitive elements. On the other hand, tRFs were found to be negatively correlated with genes that have longer exons and longer introns and a lower density in repetitive elements. These findings hold true in nearly all 32 analyzed TCGA cancers. Of note, many of the tRFs that participate in these correlations arise from mitochondrial tRNAs. This in turn raises the possibility of previously unsuspected connections among the mitochondrial genome, the nuclear genome, multiple categories of human repetitive elements, genome organization, and nuclear and mitochondrial transcription.

### Disease: tRFs as candidate liquid biopsy biomarkers

Given their presence in many settings, their multiple dependencies on tissue state, disease type and personal attributes, and their presence in circulation, tRFs are being actively explored as non-invasive biomarkers.

The presence of tRNA halves in serum was established early on (100). Soon thereafter, changes in serum tRNA halves were used to diagnose breast cancer (133), and head and neck cancer (134). Parallel work provided additional evidence that tRFs in biofluids may serve as biomarkers in several settings, including serum tRFs for cell renal cell carcinoma (135), and urine tRFs for chronic kidney disease (136), B-cell tRFs for chronic lymphocytic leukemia (130), and serum tRFs for PD (55). Of note, the PD tRF signature differs between male and female PD patients. Recently, it was also reported that the abundance in plasma of 5′-tRFs from tRNA^Gly^, tRNA^Ala^ and tRNA^Glu^ increases prior to epileptic seizures (137).

The decreasing cost of NGS is making it easier to expand these efforts to other biofluids. A recent, wide-ranging consortium study profiled many different biofluids for the presence of multiple types of short RNAs, including miRNAs and tRFs. Specifically for tRFs, the study showed that they are among the most abundant RNA types in biofluids, especially in urine (138).

It is important to note here that such differential tRF profiles are correlative in nature. While it is true that tRFs can serve as sensitive and specific biomarkers, it remains unclear whether the observed changes in tRF abundance are the cause of the disease state or result from it. Future work will need to both investigate the context in which these tRFs are produced and identify their interactions within the cell. This is analogous to the approach taken in miRNA research. To facilitate these undertakings, specialized experimental and computational tools and protocols need to be developed. We discuss these considerations next.

## TOOLS AND DATABASES

### Finding tRFs in deep-sequencing datasets

The length similarity between tRFs and miRNAs prompted several early studies to carry out tRF discovery using the same tools that were originally used for miRNAs. In several instances, sequenced reads were mapped on a small database that comprised only tRNA sequences while the rest of the genome was ignored (45,137,139–142). But as we discussed above, such approaches did not take into account the fact that the human genome contains hundreds of incomplete tRNAs in it, hundreds of sequences that resemble known tRNAs, or the fact that numerous short sequence segments exist both inside and outside of tRNA sequences.

The approach used to populate tRFdb (42), as well as tDRmapper (139), addressed several of these problems. However, neither scheme was deterministic or exhaustive. MINTmap (52) on the other hand placed emphasis on these two points guaranteeing a deterministic and exhaustive identification of tRFs in deep-sequencing datasets. To do so, MINTmap examines the whole genome in order to identify tRFs of ambiguous origin yet has minimal resource requirements (it can be run on a laptop).

### Measuring tRFs in experimental settings

tRFs present an additional complication when it comes to quantifying them in one's favorite cell line or in clinical samples. Several of the early published articles used conventional approaches such as the commercially available assays that had been developed for the study of miRNAs, including TaqMan-miRNA (41) and Exiqon-LNA (45). Several matters complicate the use of these approaches in the tRF context. First, the assays are known to be susceptible to various degrees of cross-talk by co-expressed transcripts that differ from the intended target molecule by, e.g., one or two nucleotides at either their 5′ end, 3′ end, or both. To a much lesser degree, they are also affected by co-expressed transcripts that differ in e.g. one internal position, as would be the case in instances of e.g. polymorphism or mutation. Second, these assays cannot enforce the identity of both the 5′ and 3′ endpoints of the target molecule. Third, as became evident by the large scale analysis of all of TCGA (4), multiple tRFs within a given sample have sequences that overlap extensively, differing only in their 5′ or 3′ endpoints. This is true of model cell lines as well (2). On a related note, counterpart sequence segments from isodecoders of the same isoacceptor can differ by a single nucleotide (52) making the ability to distinguish among them a relevant consideration. Lastly, the dependency of tRF production on tissue type and on personal attributes indicates that one ought to be mindful of this matter when measuring tRFs in different settings, as e.g. the tissue dependency may lead to tRFs that differ by one or two nucleotides at either endpoint (4); consequently, a different assay needs to be used each time.

We evaluated how such endpoint modifications can influence the measurements that can be obtained with commercial assays. Specifically, we designed assays for the canonical isoform of miR-21-5p (0|0) and used them to evaluate synthetic RNAs that differ at exactly one or both endpoints. While the experiments revolved around miRNA isoforms, the findings are directly applicable to tRFs. We performed the quantification assay in water and also using a model cell line. In all instances, the commercial assay was susceptible to considerable cross-talk that led to an incorrect estimate of abundance (143). Parallel studies arrived at the same conclusion (144,145).

While commercially available assays - other than NGS - cannot address the problem, a recently-reported technique provides a powerful solution to the problem. The technique, dubbed ‘dumbbell-PCR’ (146), can accurately profile isomiRs and tRFs while ensuring the identities of both terminal endpoints of the target molecule.

### Databases and servers

Analysis of all TCGA datasets uncovered more than seven thousand miRNA isoforms (147). By comparison, early efforts indicated that the number of tRFs in the same samples was going to be much higher (37). The tRFdb database (42) represents the earliest attempt to organize the tRFs that were being discovered in higher organisms into a curated repository. tRFfinder was a subsequent hybrid approach that comprised a repository of tRFs that had been discovered in TCGA and an on-line search tool that allowed web-users to search these TCGA profiles interactively (142). Subsequent analysis indicated that tRFfinder exhibited unusually low sensitivity and did not account for the numerous tRFs that were known to be produced by mitochondrial tRNAs (52).

Rigoutsos and colleagues used the MINTmap tool (see above) to profile tRFs in 12 023 human samples, from healthy donors and patients. The samples were drawn from the TCGA repository, the 1000 Genomes Project (148), and other public sources. They identified 28 824 distinct tRFs that exceed threshold in at least one of those samples. As we discussed above, whether in homeostasis, cancer, neurodegenerative disease, or viral infections, tRFs appear to serve important roles. However, of the myriad tRFs that were discovered using MINTmap, only a handful have been characterized in the literature. This makes it imperative that they be organized in a manner that facilitates their study, in the context of specific diseases and also across diseases and tissues. To this end, Rigoutsos and colleagues designed and implemented MINTbase (43,44). MINTbase is a web-based repository that permits the interactive exploration of the 28 824 human tRFs identified so far. A user can search for tRFs that are present in specific tissues, tRFs that satisfy a minimum abundance criterion, tRFs that arise from specific isoacceptors, belong to one or more of the structural categories mentioned above, etc.

## CONCLUSIONS AND PERSPECTIVE

Despite being a relative newcomer to the stage of short non-coding RNAs, tRFs have been gaining momentum for the last 10 years. Several thousand published articles later, per Google Scholar, the importance of tRFs as important players in the cell, is now well-established. However, there remains an inherent and important difficulty when it comes to studying them, which stems from several factors.

First, there is the sheer number of statistically-significant tRFs that have been discovered so far. This number already exceeds the number of protein-coding human genes. Second, their biogenesis has yet to be established and all indications are that more than one mechanisms are responsible. Third, it has been shown that tRFs, just like isomiRs, exhibit strong dependencies on variables that were not considered previously, namely, tissue, disease, and personal attributes. Essentially, a given parental tRNA isodecoder produces multiple consequential tRFs that differ from tissue to tissue, and from person to person. This complexity complicates efforts to prioritize the study of isodecoders and of the tRFs they produce. Fourth, experimenters need to cope with the constraint that quantification of individual tRFs requires more involved approaches. While there is currently one simple alternative, namely deep sequencing, it is also an expensive solution to the problem of quantifying one or only a few tRFs. Fifth, systems-level analyses have revealed links between tRFs on one hand and RNAs/proteins on the other that localize to different cellular compartments. For example, tRFs from mitochondrial tRNAs are correlated with mRNAs whose protein products are destined for the nucleus or the cellular membrane. It is unclear currently whether these correlations represent long-distance transfer of information or result from the action of translocated tRFs that interact directly with these mRNAs.

Adding to these matters are all of the findings that we enumerated above and which highlight the need to expand the traditional understanding of cancer and disease. Moreover, the time dynamics that are involved in disease development now become important. For example, we discussed how tRFs direct a temporal switch during the maturation of stem cells, slowing translation long enough for myeloid progenitors to differentiate.

In addition to mammals and plants, the findings we discussed above demonstrate important roles for tRFs in lower eukaryotes (e.g. *T. brucei*), bacteria (e.g. *S. coelicolor*) and archaea (e.g. *H. volcanii*). This is compelling evidence that tRFs hold regulatory roles in all three life domains. It is now clear that tRFs represent an elusive and, for the time being, incompletely characterized dimension of cell biology. Collectively, the findings described herein solidify tRFs as a dimension that warrants in-depth study.
